# Cigarette smoke extract affects methylation status and attenuates Sca-1 expression of mouse endothelial progenitor cell in vitro

**DOI:** 10.18332/tid/131625

**Published:** 2021-01-28

**Authors:** Zhi-Hui He, Yan Chen, Ping Chen, Li-Hua Xie, Gui-Bin Liang, Hong-Liang Zhang, Huai-Huai Peng

**Affiliations:** 1 Department of Intensive Care Unit, The Third Xiangya Hospital, Central South University, Changsha, China; 2 Department of Respiratory Medicine, The Second Xiangya Hospital, Central South University, Changsha, China; 3 Department of Respiratory Medicine, The Third Xiangya Hospital, Central South University, Changsha, China; 4 Department of Intensive Care Unit, The Second Xiangya Hospital, Central South University, Changsha, China; 5 Department of Emergency, The Second Xiangya Hospital, Central South University, Changsha, China

**Keywords:** methylation, stem cell antigen-1, dysfunction, endothelial progenitor cell, cigarette smoke

## Abstract

**INTRODUCTION:**

Endothelial dysfunction appears in many smoking-related diseases, it is also an important pathophysiological feature. Endothelial progenitor cells (EPCs) are precursors of endothelial cells and have a crucial effect on the repair and maintenance of endothelial integrity. Sca-1 is not only common in bone marrow-derived hematopoietic stem cells (HSCs), but it is also expressed in nonhematopoietic organs by tissue-resident stem and progenitor cells. The aim of this study is to investigate the impact of cigarette smoke extract (CSE) on the function of bone marrow-derived EPCs and the expression level of Sca-1 in EPCs, and also whether the methylation of Sca-1 is involved in EPC dysfunction.

**METHODS:**

We measured EPC capacities including adhesion, secretion and proliferation, the concentration of endothelial nitric oxide synthase (eNOS) and apoptosis-inducing factor (AIF) in cell culture supernatant, and also Sca-1 expression and promoter methylation in EPCs induced by CSE. Decitabine (Dec) was applied to test whether it could alter the impact caused by CSE.

**RESULTS:**

The adhesion, proliferation and secretion ability of EPCs can be induced to be decreased by CSE *in vitro*, accompanied by decreased concentrations of AIF and eNOS in cell culture supernatant and decreased Sca-1 expression in EPCs. In addition, Dec could partly attenuate the impact described above. There were no significant differences in the quantitative analysis of Sca-1 promoter methylation among different groups.

**CONCLUSIONS:**

The decreased Sca-1 expression was related to EPC dysfunction induced by CSE. EPC dysfunction resulting from CSE may be related to methylation mechanism, but not the methylation of Sca-1 promoter.

## INTRODUCTION

Cigarette smoking is regarded as a main risk factor for many diseases, especially cardiovascular and lung diseases^1^. Cigarette smoke extract (CSE) is an aqueous solution containing most of the compounds and toxins inhaled by smokers, and commonly used as a preferred alternative for cigarette smoke (CS)^2^. CSE has been used in cell experiments *in vitro*, and has been proven to have a direct effect on cell dysfunction^3^. Researchers initially isolated the endothelial progenitor cells (EPCs) in 1997^4^. EPCs derived from bone marrow are the primary origin of endothelial cells and have a crucial effect on major vasculogenic tissues of the body including heart and lung^5^. EPCs repair tissues through pivotal bioactivities including secretion of vasoactive substances, migration, homing and proliferation^6^. Our previous studies have shown that in patients who suffer from chronic obstructive pulmonary disease (COPD), the number of circulating EPCs is decreased and their function is out of order^7^ compared with healthy people. There is evidence that EPCs play an important role in the occurrence and development of COPD^8-12^. But, the mechanism how cigarette smoke influences EPC function remains unknown.

Endothelial nitric oxide synthase (eNOS), the key enzyme for catalyzing the production of NO, could maintain vascular homeostasis by hypoxic compensatory mechanism, tension regulation, vascular permeability and blood pressure homeostasis^13^. Our previous study demonstrated that eNOS could maintain EPC homeostasis and function^14^. Apoptosis-inducing factor (AIF), a mitochondrial intermembrane space protein, has the ability to maintain electron transport chain function, regulate reactive oxygen species (ROS), cell death, and neurodegeneration^15^. AIF can also protect the rapidly proliferating cells from death induced by ROS^16^.

Sca-1, found on hematopoietic stem cells (HSCs), is a glycosylphosphatidylinositol anchored protein and is considered to be a cell surface marker^17^. Sca-1 can promote adhesion and proliferation of EPCs, which play a key role in optimal hematopoietic activity^18^. Upregulated Sca-1 level in EPCs could improve cardiac function recovery from myocardial infarction in rats^19^, indicating to some extent that Sca-1 is directly or indirectly involved in the regulation of EPCs.

Methylation is a common epigenetic mechanism, which can transfer a methyl group to the C5 position of cytosine^20^. It participates in regulation of gene expression, gene silence, DNA damage reparation, and other important biological processes^21^. Methylation is one of the epigenetic modifications. It exists widely and affects directly DNA molecules in eukaryotes. In mammals, it almost exclusively occurs in the context of CG dinucleotides^22^. From the bioinformatics directly we can ascertain if there are CG dinucleotides in the Sca-1 promoter sequence (http://www.urogene.org/ methprimer/). In terms of previous experiments, it was suggested that the regulation of methylation may participate in Sca-1 expression^23^. Decitabine (Dec) is a demethylation reagent and widely used in medical treatment, it can activate demethylation *in vitro* and *in vivo* ^24^ and make epigenetic silencing genes reactivate again^25^. In this experiment, we explored the changes of EPC function, the concentrations of eNOS and AIF in cell culture supernatant, Sca-1 expression, and the changes of Sca-1 promoter methylation in EPCs induced by CSE, and whether the dysfunction of EPCs induced by CSE is related to Sca-1 promoter methylation.

## METHODS

### Subjects

The EPCs used in this experiment were derived from healthy C57BL/6J mice 4–6 weeks old. Animals were offered by Shanghai Laboratory Animal Center (Shanghai, China) and raised in a chamber with 50–60% humidity at 23–25°C while being subjected to 12-hour circadian rhythm. The study was approved by Institutional Review Board of Central South University, in keeping with the EU Directive 2010/63/EU^26^.

### Obtention of EPCs

EPCs are derived from mouse bone marrow, as previously described^27^. Simply, mononuclear cells were isolated from bone marrow, inoculated in a culture bottle, incubated with EGM-2 (Lonza, Switzerland), and placed in an atmosphere at 37°C, 95% humidity and 5% CO_2_. On the 4th day of incubation, we replaced the old medium with fresh medium, and removed the cells that became unattached to the wall. Every three days, we removed half of the old medium and replace it with a fresh one. Cells were collected on the 7th day of culture.

Three methods were applied to identify the EPCs^27^: morphology of EPCs, double staining with 1,1’-dioctadecyl-3,3,3’,3-tetramethylindocarbocyanine perchlorate-labeled acetylated low-density lipoprotein (Dil-acLDL from Eugene, USA) and fluorescein isothiocyanate-labeled Ulex europaeus agglutinin-1 (FITC-UEA-1 from Sigma, USA), and co-expressing of FITC-CD34 + / PE-CD133 + / APC-Flk-1 + (FITC-CD34 and APC-Flk-1 from Becton Dickinson, USA; PE-CD133 from eBioscience, USA). PerCP-conjugated anti-mouse Sca-1 antibody (PerCP-Sca-1 from Biolegend, USA) was used to detect Sca-1 positive rate.

### CSE and DEC solution

The CSE was acquired as described previously^14^. Briefly, we burned one cigarette (carbon monoxide 14 mg/cigarette, nicotine 1 mg, tar 13 mg; Tobacco Hunan Industrial, China), collected the smoke in 20 mL of EGM-2 by a vacuum pump, removed particles and bacteria by 0.22 μM pores, then diluted the solution to 1% CSE with EGM-2. In 2 mL of EGM-2, we dissolved 5 g of Dec (Sigma, California, USA), then diluted the solution to 2 μmol/L with EGM-2 and stored it at -80°C until the experiments.

### Test on the capacities of EPCs

The EPCs on day 7 of the culture were trypsinized (0.25% trypsin from Amresco, Cleveland, OH, USA), resuspended with EGM-2 and transplanted to a 96-well plate (1×10^4^ in 200 μL per well). The wells were divided into 3 groups: control group, CSE group, and CSE+Dec group. We added 200 μL EGM-2 per well to the control group and CSE group, and 200 μL Dec solution per well to the CSE+Dec group. After being incubated for 48 h, the culture media of the cells were removed and replaced by 200 μL EGM-2 per well in the control group, and by 200 μL CSE solution per well in the CSE group and CSE+Dec group. After being incubated for 24 h, the EPCs were used for the following assays. The test on the capacities of EPCs was conducted as described previously^14^. The proliferation capacity of EPCs was measured by 3-(4,5-dimethylthiazol-2-yl)-2,5-diphenyltetrazolium bromide (Sigma, California, USA) assay, the adhesive capacity was assessed by adherent cell count, and the secretion capacity was measured by the concentration of NO in EPC culture fluid.

### Concentrations of eNOS and AIF in cell culture supernatants

According to the instructions, the concentrations of eNOS and AIF in the supernatant of cell culture were determined with ELISA kits (R&D systems, USA).

### Detection of protein expression

Briefly, EPCs were washed, lysed and centrifuged. The protein was blended with 2×SDS loading buffer (1:1), incubated at 100°C for 4 min, then electrophoresed in 12% SDS-polyacrylamide gel and transferred to polyvinylidene difluoride microporous membrane (Millipore, Massachusetts, USA) by electrophoresis. Next, membranes were incubated with 1:200 primary antibody (Biolegend, USA) overnight, then re-incubated with secondary antibody (1:3000) for 1 h after being washed. After being washed again, the membranes were detected.

### Detection of RNA expression

Trizol (Invitrogen, California, USA) was used to extract RNA from EPCs. RevertAid™ First Strand cDNA Synthesis Kit (Fermentas, USA) was used to synthesize the first strand of cDNA which was regarded as template for quantitative RT-PCR (SYBR Green qPCR Master Mix, USA) with an internal control of β-actin. The fold change of expression level was analyzed using ΔΔCt method. The primers’ sequences were:

Sca-1, 5'-ACACCGAGCCCAGGTAACCC-3' (forward) and 5'-CTGGTCCGCTCAGGACAGCA-3' (reverse) (Primer 5); β-actin, 5'-CATCCTGCGTCTGGACCTGG-3' (forward) and 5'-TAATGTCACGCACGATTTCC-3' (reverse).

### Methylation of Sca-1 promoter

The methylation of Sca-1 promoter was detected by bisulfite sequencing PCR (BSP)^28^. Briefly, genome DNA extraction kit (Takara, Japan) was used to extract genome DNA from EPCs. Sodium bisulfite conversion of genomic DNA was carried out with EpiTect Bisulfite Kit (Qiagen, Dusseldorf, Germany). Primers sequences for BSP synthesized by Genscript Co., Ltd (Nanjing, China) were as follows: 5'-TGGTAGGGTTTATTATTTGGAT-3' (forward) and 5'-CTCACAAAACAACTAAATCCCA-3' (reverse).

The product of PCR was then purified and cloned into pMD18-T vector. The sequence of product was analyzed on Applied-Bio 3730 (Applied-Bio, Carlsbad, California, USA). The methylation rate (%) was calculated from:

Methylation rate = amount _methylatedCpG_ / (amount_samples_×amount_clones_×amount_CpG_)×100%.

### Statistical analysis

Each experiment was repeated three times. SPSS 16.0 (SPSS Inc., Illinois, USA) was used for the analyses. Data presented are expressed as mean ± standard deviation (SD). One-way ANOVA was used to analyze the differences among groups, *post hoc* analysis was carried out as appropriate. Statistical significance was defined as p<0.05.

## RESULTS

### Obtention of EPCs

During culture, the morphology of EPCs changed noticeably. First, cells were suspended in the media with the same shape and size (Supplementary file, Figure 1A). Gradually, the cells became oval, spindle, enlarged and tending to attach to each other and form ball-like structures (Supplementary file, Figure 1B). After a few more days, cells were fusiform, polygonal, in contact with each other and forming capillary structures (Supplementary file, Figure 1C). Analysis of double staining of Dil-acLDL and FITC-UEA-1 showed that the rate of amphoteric cells was 98.0% (Supplementary file, Figure 2).

**Figure 1:**
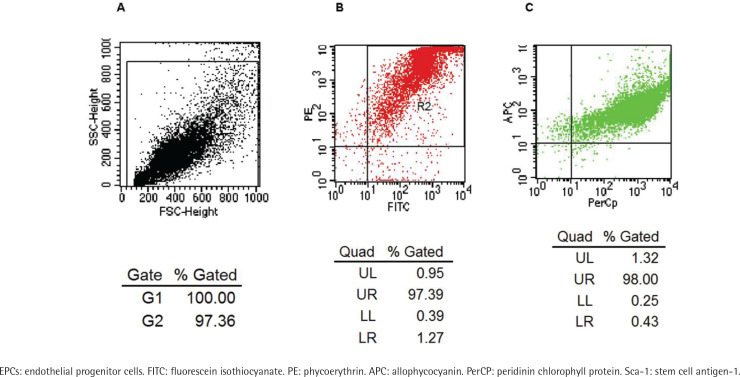
Identification of EPCs by co-expression with FITC-CD34, PE-CD133, APC-Flk-1 and PerCP-Sca-1. Flow cytometry scatter plot of the test cells is shown in Panel A. The rate of the cells co-expressing FITC-CD34 and PE-CD133 was 97.39% (Panel B). The rate of the cells which were derived from panel B co-expressing APC-Flk-1 and PerCP-Sca-1 was 98% (Panel C).

A flow cytometry scatter plot of the test cells is shown in Figure 1A. The FACS test showed that most cells co-expressed PE-CD133 and FITC-CD34 (Figure 1B), while most of the cells isolated from Figure 1B co-expressed APC-Flk-1 and PerCP-Sca-1 (Figure 1C). The positive rate of co-expression of PE-CD133, FITC-CD34 and APC-Flk-1 in cells, which were regarded as EPCs, was 97.39 × (1.32 + 98) = 96.73%. The positive rate of Sca-1 on EPCs was 97.39 × 98 = 95.44%.

### The function of EPCs

The OD value of EPCs in CSE+Dec group and CSE group was significantly lower than in the controls. The OD value of EPCs in CSE+Dec group was significantly higher than that in CSE group (Supplementary file, Figure 3A). The secretion and adhesion of EPCs in CSE+Dec group and CSE group were significantly lower than in the controls. Further, the adhesion and secretion of EPCs in CSE+Dec group were significantly higher than those in CSE group (Supplementary file, Figure 3B).

### The concentrations of eNOS and AIF in cell culture supernatant

**Figure 2:**
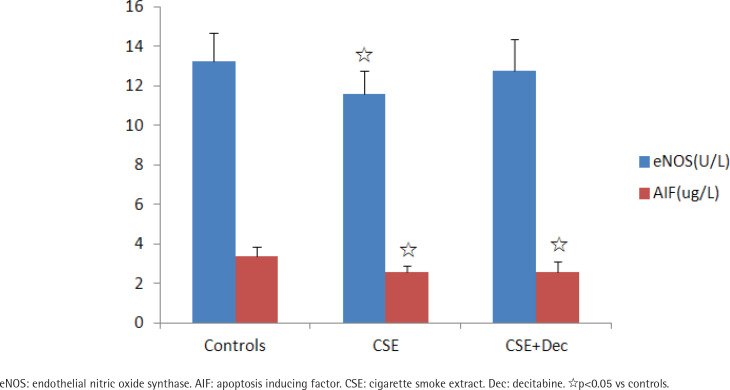
The concentrations of eNOS and AIF in cell culture supernatant. Data are presented as mean ± SD.

The concentration of eNOS in CSE group was significantly lower than in the controls. There was no difference in the concentration of eNOS between CSE+Dec group and CSE group, as well as between CSE+Dec group and controls. The concentrations of AIF in CSE group and CSE+Dec group were significantly lower than in the controls. But there was no difference in the concentration of AIF between CSE+Dec group and CSE group (Figure 2).

### Sca-1 expression in EPCs

**Figure 3:**
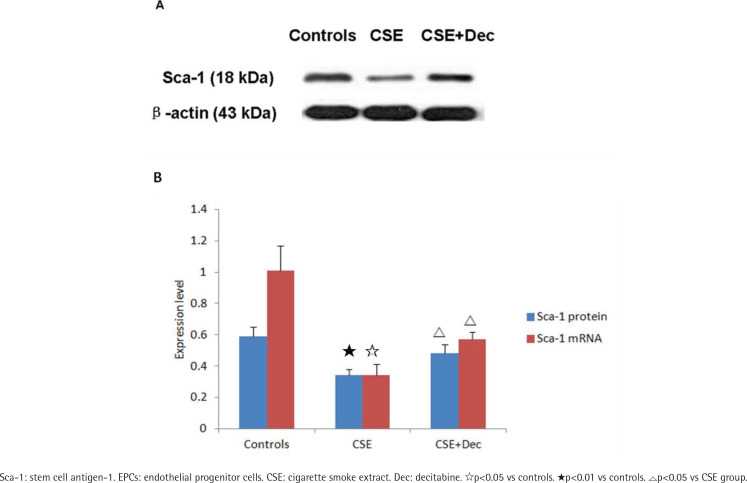
The expression levels of Sca-1 protein and mRNA in EPCs. Data are presented as mean ± SD.

In EPCs, the expression level of Sca-1 protein and mRNA in CSE group were significantly lower than in CSE+Dec group or controls (Figures 3A and 3B). There was no difference in Sca-1 protein or mRNA between CSE+Dec group and controls.

### The changes of Sca-1 promoter methylation

**Figure 4:**
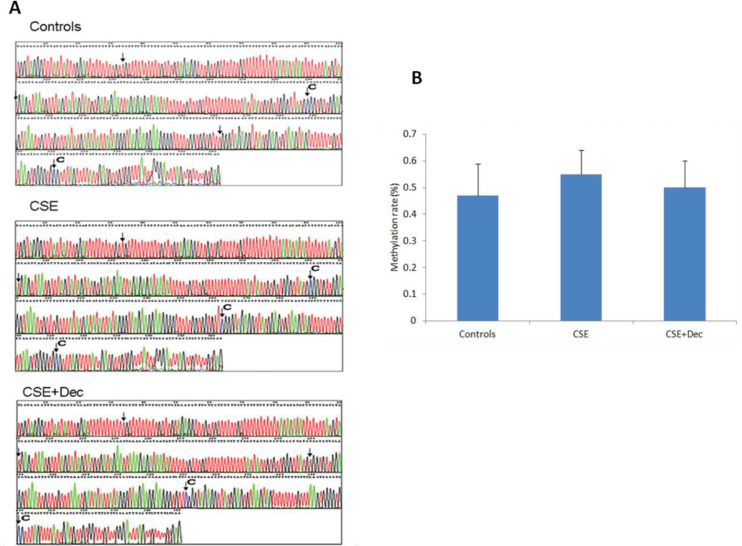
The changes of Sca-1 promoter methylation. The arrows in the pictures indicate the site of CpG dinucleotides, and the symbol of ‘C’ indicates the site of methylation (Panel A). There was no significant difference in quantitation among the three groups (Panel B). Data are presented as mean ± SD.

The representative methylation maps are shown in Figure 4A. The arrows in the pictures indicated the site of CpG dinucleotides, and the symbol of ‘C’ indicates the site of methylation. Quantitative analysis on methylation rate of CpG dinucleotides showed no difference in Sca-1 promoter methylation among controls, CSE+Dec group and CSE group (Figure 4B).

## DISCUSSION

We found that CSE-induced EPCs exhibited noticeably declined capacities of proliferation, secretion and adhesion, which were accompanied by declined concentrations of eNOS and AIF in cell culture supernatant and declined Sca-1 expression level. Dec could partly alleviate the changes described above. However, there was no significant difference in the quantitative analysis of Sca-1 promoter methylation among different groups. These results strongly imply that CSE induces the dysfunction of EPCs through methylation mechanism. Dec could partly protect against the dysfunction of EPCs through demethylation of the upstream gene or transcription gene of Sca-1, upregulating Sca-1 expression. Although the result of Sca-1 promoter methylation was negative, it is also of value.

We identified EPCs by morphology, double staining with FITC-UEA-1 and Dil-acLDL, and the detection of surface markers including CD133, CD34 and Flk-1 by FACS. Indeed, we obtained highly purified EPCs. The results confirmed that there was a high positive rate of Sca-1 on EPCs. As the precursor of endothelial cells, EPCs with normal abilities of secretion, proliferation, self-renewal and differentiation are necessary for their own shift of trans-endothelia, homing onto an injured site, repair and regeneration of organs and tissues^29^. EPC dysfunction observed in this experiment was consistent with what was observed in patients with chronic obstructive pulmonary disease (COPD)^7^.

NO derived from eNOS could be used as vascular endothelial regulator, antioxidant platelet inhibitor and endogenous vasodilator^30^. eNOS was first suggested as a constitutive enzyme, but actually it was the NOS isoform in the vasculature^31^. One study found that the activation of the Akt/eNOS/NO pathway attenuated HG-induced EPC dysfunction^32^. Another study observed that reverse-D-4F reversed the dysfunction of lipopolysaccharide-induced EPCs partially through PI3K/AKT/eNOS signaling pathway^33^. Du et al.^34^ found that eNOS signaling activity was downregulated in transient receptor potential-canonical 1 knockout mice, which reduced the migration and tube formation of EPCs. Wei et al.^35^ reported that ZFP580 could increase the expression of eNOS and the availability of nitric oxide to promote the differentiation of EPCs into ECs. In the present study, the eNOS expression in CSE group was decreased, indicating that the decreased eNOS caused by CSE was involved in the dysfunction of EPCs.

AIF is widely expressed in all cell lineages and plays the role of NADH oxidase in mitochondria^36^. AIF contributes to caspases-independent cell apoptosis and participates in the assembly of cell respiratory chain^37^. There is evidence that AIF can transfer from mitochondrion to nucleus during virus infection, and AIF inhibitors can significantly reduce apoptosis^38^. Smoking exposure leads to AIF translocation to mitochondrial nucleus in bronchial epithelial cells of humans, which increases the activation of smoking mediated parthanatos pathway and leads to smoke-related lung diseases^39^. Palmitoyl ceramide significantly reduced cellular proliferation and apoptosis via AIF translocation in human lung cells^40^. Retroviral insertion into the gene could reduce AIF expression and oxidative phosphorylation in retina and brain of mice, which correlates with retinal degeneration and neuronal defects^41^. In muscle specific AIF knockout mice, the protein expression and activity of respiratory chain complex I were decreased and damaged. Mice will develop skeletal muscle atrophy, severe dilated cardiomyopathy, and even heart failure^42^. The study of van Empel et al.^43^ found that AIF deficiency resulted in higher sensitivity to oxidative stress and necrotic-like cell death in cardiomyocytes. In this experiment, the concentrations of AIF in cell culture supernatant were significantly reduced after CSE intervention, suggesting that the reduced AIF participated in the EPC dysfunction resulting from CSE. The decrease in AIF may be the result of CSE induced mitochondrial nuclear translocation of AIF, which needs further study.

Sca-1 is a member of *Ly6* polygenic family, which encodes highly homologous glycosyl-phosphatidylinositol-anchored membrane proteins and is the most common marker for enriching adult HSCs^44^. Hyperbaric oxygen could up-regulate Sca-1 level of EPCs to improve outcome of critical limb ischemia in rats^45^. Combination of telmisartan and simvastatin could increase the number of EPCs through enhancing Sca-1 expression^46^. Shmilovich et al.^47^ found that systemic B-type natriuretic peptide (BNP) administration to mice could make Sca-1 levels significantly increased in bone-marrow-derived EPCs and improve blood flow in the ischemic limbs of mice.

Everaert et al.^48^ reported that the deficiency of adiponectin, a protein with fat-linking function, reduced the concentration and mobilization of Sca-1 in progenitor cells, which reduced the vascular adhesion molecules and hypoxia-induced chemokines expression, and ultimately damaged the ability of neovascularization. In this experiment, the change of Sca-1 expression was consistent with the changes of EPC functions, which suggested that Sca-1 may be at least in part responsible for EPC function, and thereby for the repair of endothelium. The results of this experiment may be helpful in understanding the vascular microstructural damage in smoke-related diseases.

Methylation is an important epigenetic modification, which modifies the methyl groups in gene without changing gene sequence of bases^49^. Downregulating the level of methylation or demethylation could activate gene expression, leading to up-regulation of oncogene expression or making the chromosome unstable^50^. Upregulating the level of methylation could turn off the activity of some genes and affect DNA repair^51^. Hyperhomocysteinemia could induce the dysfunction of EPCs through up-regulating the methylation level of Cystathionine-β-synthase promoter^52^.

Methyl-CpG-binding protein 2 could reduce the angiogenesis of aging EPCs and promote apoptosis through hypermethylation of sirtuin 1 promoter, leading to EPC aging and dysfunction^53^. There is little research on the methylation of Sca-1. There was a study that implied that with decreasing methylation level of Wnt inhibitory factor 1, the efficiency of differentiation of Sca-1+ adult cardiac progenitor cells *in vitro* increases^54^. The present study indicated that there was no difference in the quantitative analysis of Sca-1 promoter methylation among different groups, which did not exactly match with the results that there were significant differences in Sca-1 expression and EPC functions among different groups. The reasons for the inexact match may lie in that Dec is a non-specific demethylation agent, and its affect on other genes could not be ruled out. So, the issue of whether there is methylation or demethylation in upstream gene or transcription gene of Sca-1 deserves further study.

## CONCLUSIONS

This study confirmed that Sca-1 was involved in EPC dysfunction caused by CSE and that Dec could partly protect against the dysfunction of EPCs by improving Sca-1 expression, which may be the cell basic of smoke-related vasculogenic diseases. Although the result of Sca-1 methylation was negative, it is also of value. It is possible that the methylation or demethylation of upstream gene or transcription gene of Sca-1 may participate in EPC dysfunction caused by CSE, which deserves further study.
