# Supplementary material for: Cigarette smoke extract affects methylation status and attenuates Sca-1 expression of mouse endothelial progenitor cell in vitro
Source: Tob Induc Dis. 2021 Jan 28;19:08. doi: 10.18332/tid/131625 (PMC7842580; doi:10.18332/tid/131625)
Supplement: Supplementary file 1 [file TID-19-08-s1.pdf]

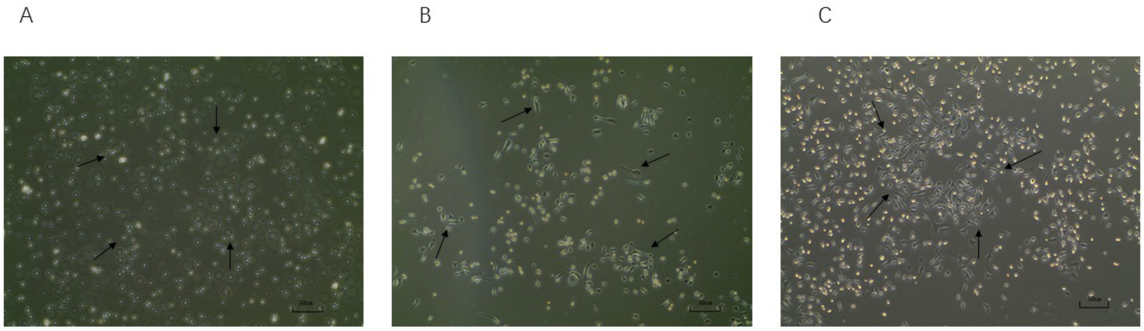

Figure 1. The morphological change of EPCs sourced from bone marrow during incubation. On day 1 of the incubation, cells were round in shape and suspended in media (Panel A). On day 4 of the incubation, cells were oval, spindle or polygonal in shape and attempted to attach to each other (Panel B). On day 7 of the incubation, cells grew up to fusiformis or polygon and contacted with each other to form capillary structure (Panel C). The scale bar represents 50 µm. EPCs: endothelial progenitor cells.

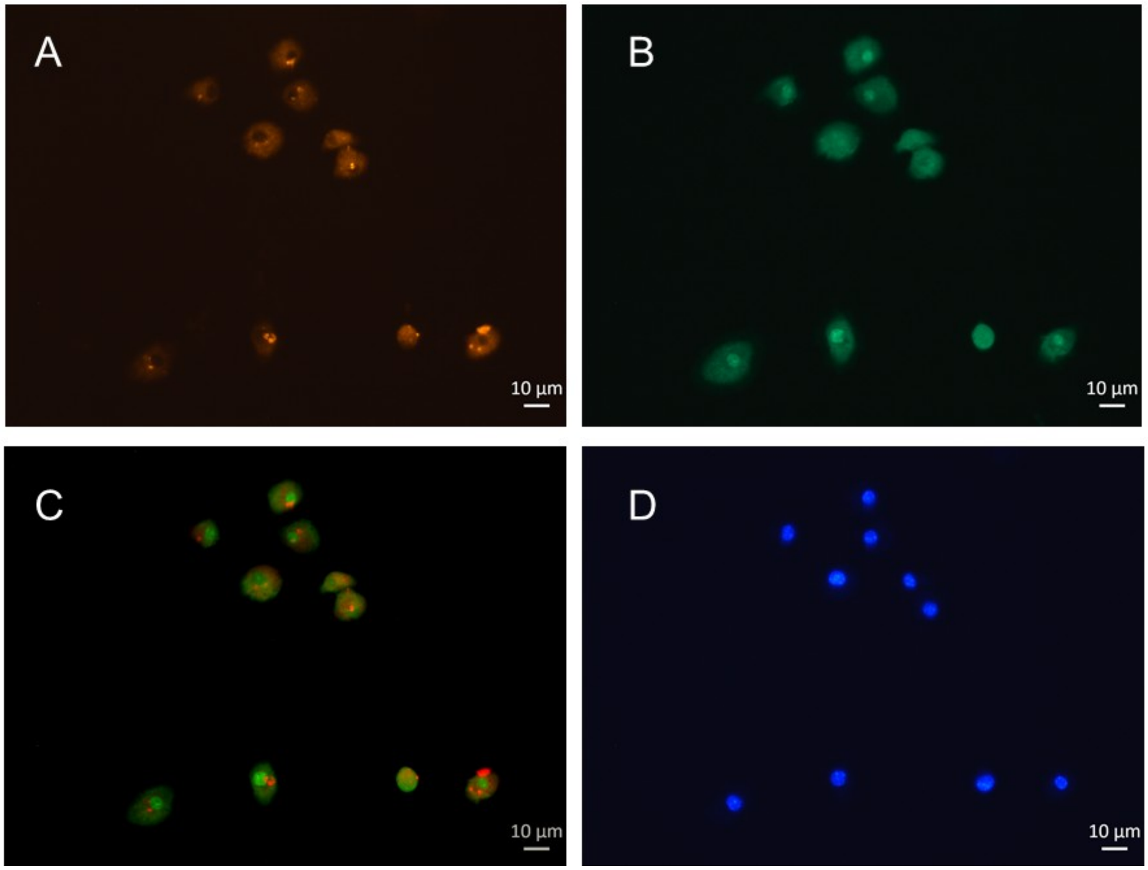

Figure 2. Identification of EPCs by double positive staining with Dil-acLDL and FITC-UEA-1. By laser scanning confocal microscope (LSCM), it was demonstrated that the cells displayed red cytoplasm while taking up Dil-acLDL on day 7 of the incubation (Panel A), green cytomembrane when binding FITC-UEA-1 (Panel B), and orange when positively stained with Dil-acLDL and FITC-UEA-1 (Panel C), and blue when staining with DAPI in nuclear localization (Panel D). The scale bar represents 10 μm.

Note: EPCs, endothelial progenitor cells; Dil, 1,1'-dioctadecyl-3,3,3',3-tetramethylindocarbocyanine perchlorate; acLDL, acetylated low density lipoprotein; FITC, fluorescein isothiocyanate; UEA-1, ulex europaeus agglutinin-1; DAPI, 4',6-diamidino-2-phenylindole.

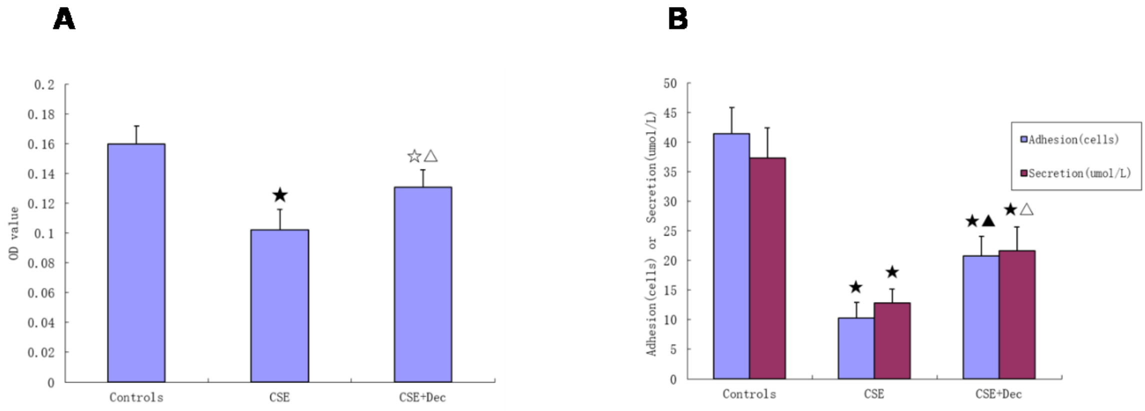

Figure 3. The comparisons of EPC functions. They are graphs of quantitative analysis of proliferation (Panel A), and adhesion and secretion (Panel B) of EPCs in different groups. Data are represented as mean ± SD.

EPCs: endothelial progenitor cells. Dec: decitabine.

☆: p<0.05 vs controls; ★: p<0.01 vs controls. △: p<0.05 vs CSE group. ▲: p<0.01 vs CSE group.

© 2021 He Z.H. et al.
